# Supplementary material for: Extreme warming challenges sentinel status of kelp forests as indicators of climate change
Source: Nat Commun. 2016 Dec 13;7:13757. doi: 10.1038/ncomms13757 (PMC5159872; doi:10.1038/ncomms13757)
Supplement: Supplementary Information — Supplementary Figures 1-5 and Supplementary Tables1-2. [file ncomms13757-s1.pdf]

Supplementary Figures

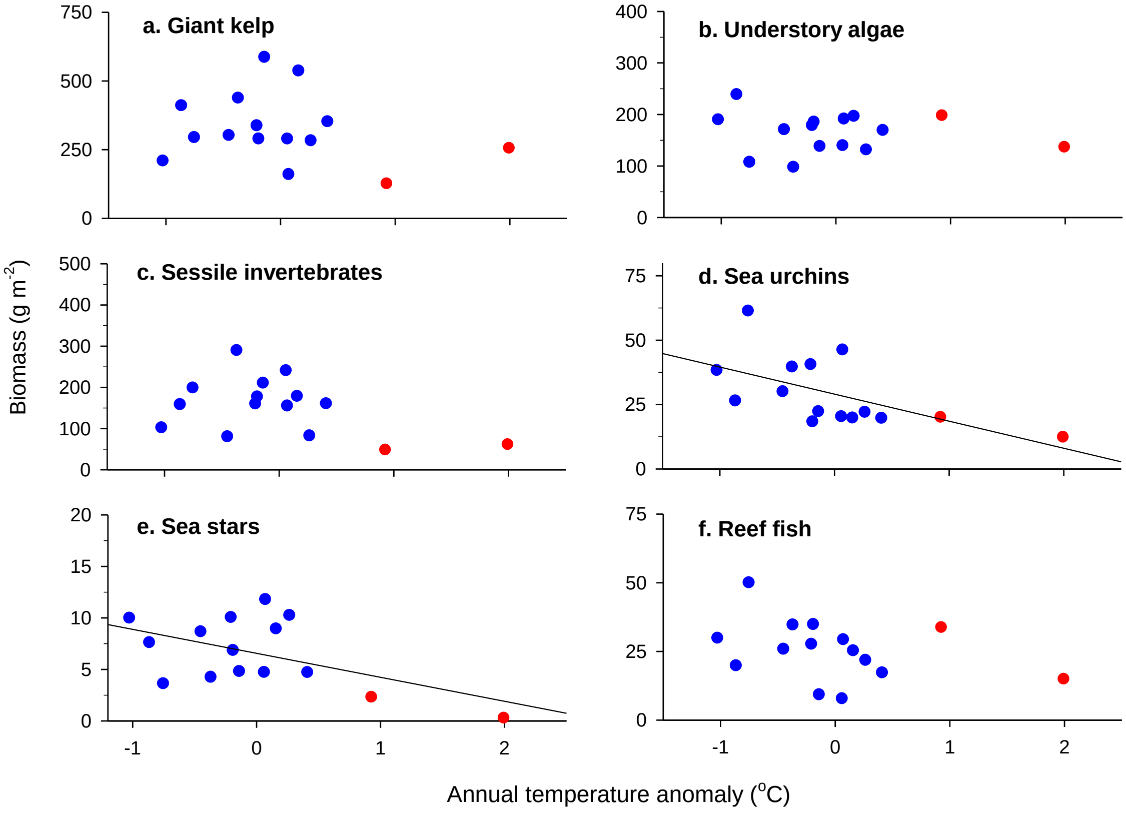

Supplementary Figure 1 Ecosystem responses to bottom temperature anomalies. Biomass values are annual means measured in summer averaged over nine sites and are in units of decalcified dry mass except reef fish, which are in units of dry mass. Values for bottom temperature anomaly represent the 12-month period preceding the summer measurements of biomass. Values for 2001-2013 are shown in blue and 2014-2015 in red. Regression lines are provided for cases where temperature anomaly contributed to a significant model.

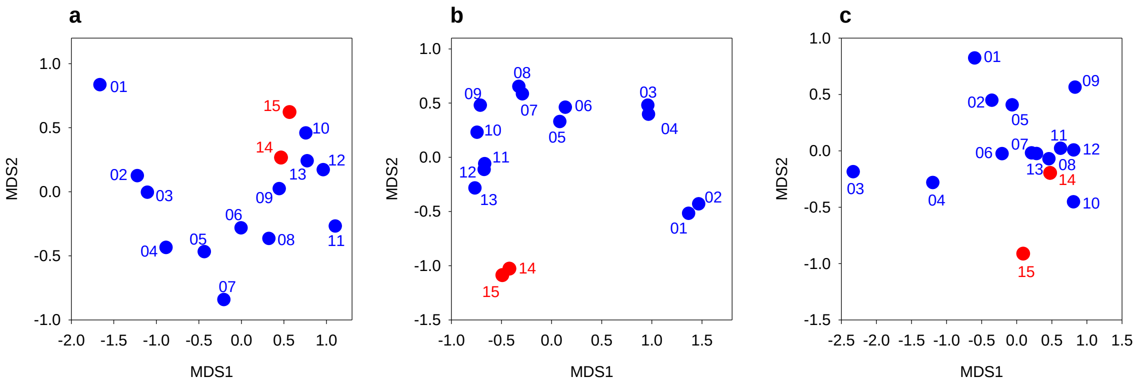

Supplementary Figure 2 Species composition responses to anomalous warming. Multi-dimensional scaling plots comparing the species assemblages of (**a**) understory algae, (**b**) sessile invertebrates and (**c**) reef fishes for cool (blue) and warm (red) periods. Numbers next to points indicate year. Differences in assemblage structure as detected by PERMANOVA were as follows: understory algae Pseudo-$F_{1,13}$ = 1.34, P(perm) = 0.283; sessile invertebrates Pseudo-$F_{1,13}$ = 2.86, P(perm) = 0.049; reef fish Pseudo-$F_{1,13}$ = 0.99, P(perm) = 0.384.

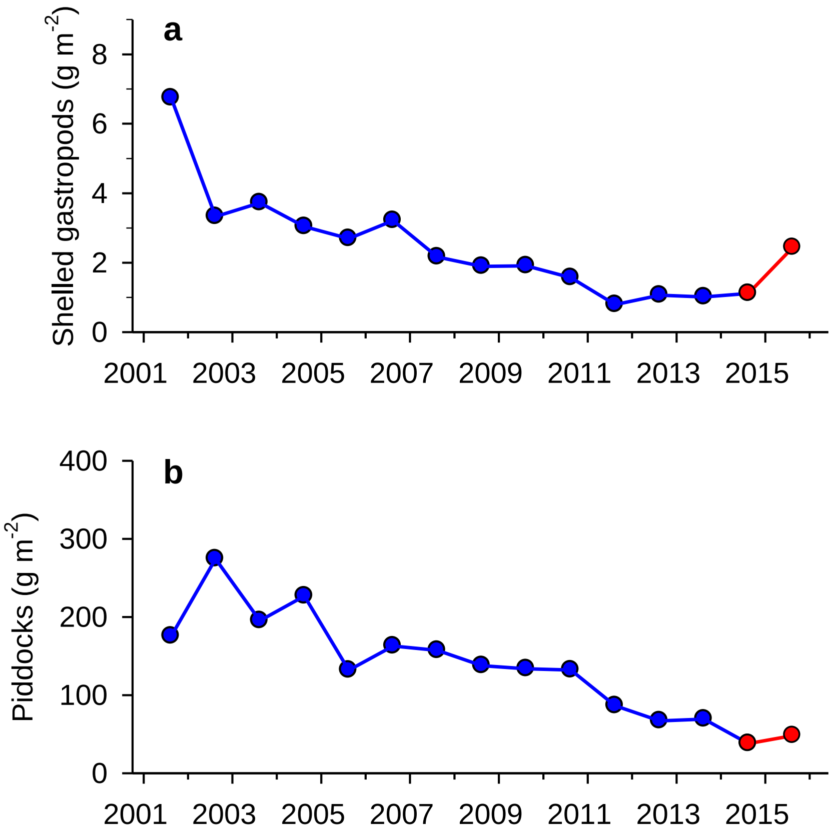

Supplementary Figure 3 Mean biomass of major prey items of sea stars. (**a**) shelled gastropods and (**b**) piddocks (Pholadidae). Blue indicates cool period (2001 -2013) and red indicates warm period (2014-2015). Biomass is in units of decalcified dry mass.

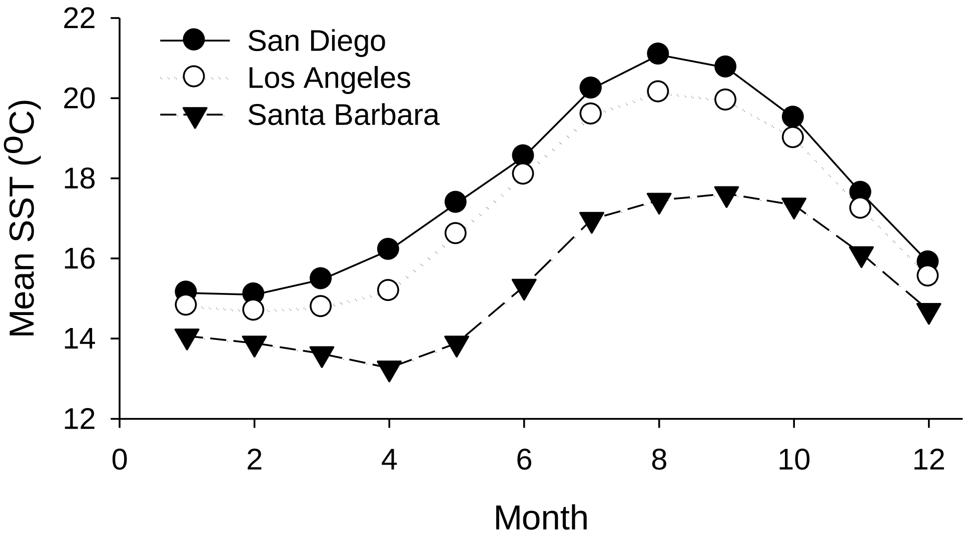

Supplementary Figure 4 Sea surface temperatures (SST) in southern California. Mean monthly SST averaged over the period 1980 – 2015 for coastal waters offshore of the counties of (south to north) San Diego, Los Angeles and Santa Barbara.

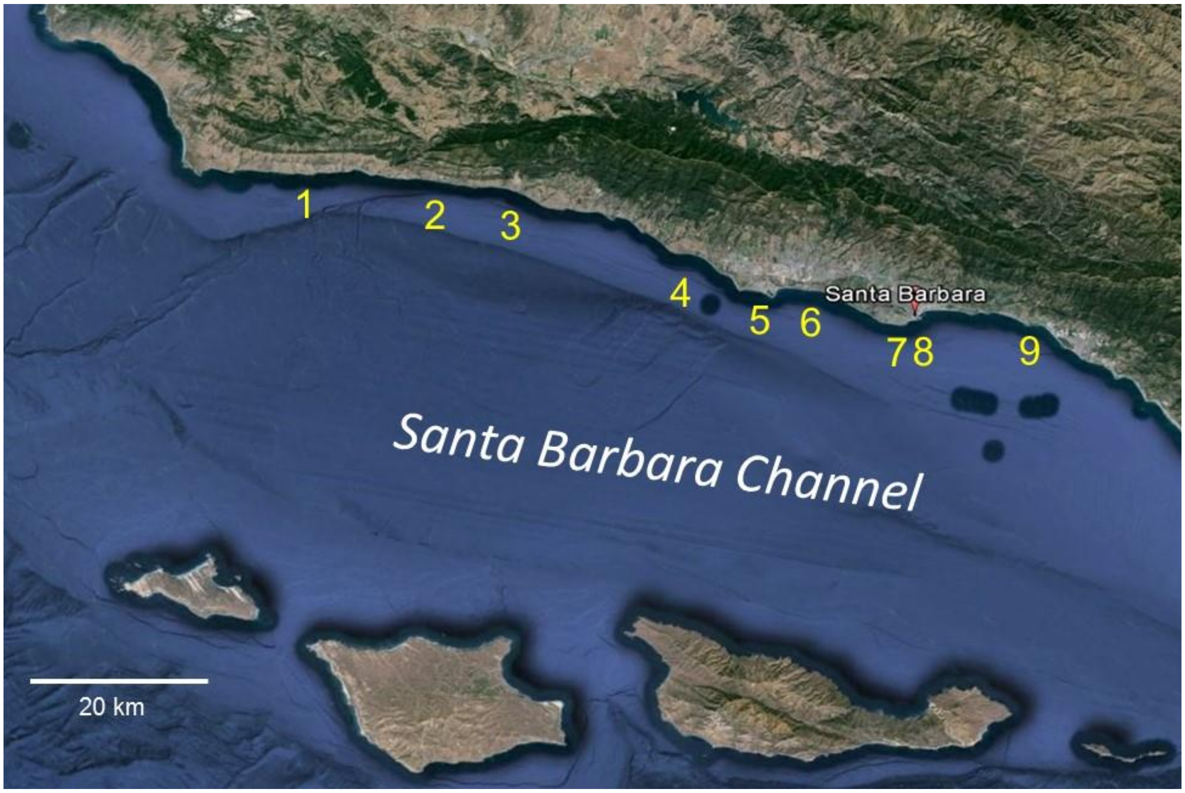

Supplementary Figure 5 Study site locations. Google map of the Santa Barbara Channel in southern California showing the locations of the Santa Barbara Coastal Long Term Ecological Research program's long-term kelp forest sites. 1. Bulito (34.45851 N, -120.33349 W); 2. Arroyo Hondo (34.47182 N, -120.14261 W); 3. Arroyo Quemado (34.46775 N, -120.11905 W); 4. Naples Reef (34.42212 N, -119.95154 W); 5. Isla Vista (34.40278 N, -119.85755 W); 6. Goleta Bay (34.41372 N, -119.82210 W); 7. Arroyo Burro (34.40028 N, -119.74459 W); 8. Mohawk (34.39407 N, -119.72957 W); 9. Carpinteria (34.39163 N, -119.54169 W).

Supplementary Table 1 Results of Simper analysis showing taxon-specific differences in the percent cover of sessile invertebrates between the cool (2001-2013) and warm (2014-2015) periods. Individual and cumulative Bray-Curtis dissimilarity values for the species most responsible for differences in assemblage structure between the cool and warm periods are provided. Average Bray-Curtis dissimilarity between periods was 21.3%.

	**Mean percent cover**		**Bray Curtis Dissimilarity**	
Taxon	2001-2013	2014-2015	% Contribution	% Cumulative contribution
Polyclinum planum	10.14	0	4.18	4.18
Leucilla nuttingi	9.63	0	4.02	8.20
Eudistylia polymorpha	5.74	13.34	3.88	12.08
Pachythyone rubra	8.09	16.63	3.84	15.92
Pholadidae spp.	0	8.69	3.62	19.54
Chelyosoma productum	7.86	0	3.29	22.83
Balanus spp.	6.28	13.49	3.26	26.09
Pachycerianthus fimbratus	13.83	6.48	3.17	29.26
Muricea fruticosa	8.91	6.55	2.93	32.19
Serpulorbis squamigerus	2.70	9.23	2.77	34.96
Watersipora subatra	0	6.32	2.69	37.65
Cucumeria piperata	6.46	0	2.68	40.33
Pycnoclavella stanleyi	8.06	4.87	2.65	42.98
Aglaophenia spp.	9.69	3.79	2.49	45.47
Salmacina tribranchiata	5.31	5.40	2.32	47.79
Cellaria sp.	3.19	7.84	2.29	50.08
Metridium senile	0.97	6.01	2.23	52.32
Euherdmania claviformis	7.28	10.16	2.21	54.53
Bryozoa spp.	5.13	0	2.18	56.71
Acanthancora cyanocrypta	3.48	4.15	1.89	58.60
Lissothuria nutriens	4.80	3.89	1.78	60.39
Pista elongata	4.37	0	1.78	62.16
Cirriformia luxuriosa	3.24	6.60	1.76	63.92
Hipporina mexicana	6.57	8.11	1.73	65.65
Plumularia spp.	4.12	0.5	1.61	67.27
Archidistoma psammion	11.84	9.66	1.60	68.87
Clavelina spp.	3.31	2.61	1.47	70.34

Supplementary Table 2 Kelp forest taxa sampled by the Santa Barbara Coastal Long Term Ecological Research program that are included in: (1) Understory algae, (2) Sessile suspension feeding invertebrates, (3) Sea urchins, (4) Sea stars and (5) Reef fish.

1. UNDERSTORY ALGAE

TAXON	PHYLUM
Cladophora graminea	Chlorophyta
Codium fragile	Chlorophyta
Filamentous green spp.	Chlorophyta
Ulva spp.	Chlorophyta
Benthic diatom	Chromista
Colpomenia spp.	Phaeophyta
Desmarestia ligulata	Phaeophyta
Dictyopteris undulata	Phaeophyta
Dictyota spp.	Phaeophyta
Eisenia arborea	Phaeophyta
Filamentous brown spp.	Phaeophyta
Laminaria farlowii	Phaeophyta
Pterygophora californica	Phaeophyta
Sargassum horneri	Phaeophyta
Sargassum muticum	Phaeophyta
Scytosiphon lomentaria	Phaeophyta
Stephanocystis osmundacea	Phaeophyta
Taonia lennebackerae	Phaeophyta
Acrosorium ciliolatum	Rhodophyta
Anisocladella pacifica	Rhodophyta
Bladey red spp.	Rhodophyta
Bossiella orbigniana	Rhodophyta
Branching red spp.	Rhodophyta
Calliarthron cheilosporides	Rhodophyta
Callophyllis flabellulata	Rhodophyta
Chondracanthus spinosa	Rhodophyta
Chondracanthus spp.	Rhodophyta
Corallina chilensis	Rhodophyta
Cryptopleura ruprechtiana	Rhodophyta
Cryptopleura spp.	Rhodophyta
Encrusting coralline spp.	Rhodophyta
Encrusting fleshy red spp.	Rhodophyta

Fauchea spp.	Rhodophyta
Filamentous red spp.	Rhodophyta
Red turf	Rhodophyta
Gelidium robustum	Rhodophyta
Gelidium spp.	Rhodophyta
Gracilaria spp.	Rhodophyta
Gymnogongrus spp.	Rhodophyta
Halosaccion glandiforme	Rhodophyta
Iridaea spp.	Rhodophyta
Laurencia spp.	Rhodophyta
Lithothrix spp.	Rhodophyta
Neoagardhiella baileyi	Rhodophyta
Nienburgia andersoniana	Rhodophyta
Osmundea spectabilis	Rhodophyta
Phycodrys setchelli	Rhodophyta
Polyneura latissima	Rhodophyta
Prionitis angusta	Rhodophyta
Prionitis lanceolata	Rhodophyta
Rhodymenia californica	Rhodophyta
Sarcodiotheca furcata	Rhodophyta
Sarcodiotheca gaudichaudii	Rhodophyta
Scinaia confusa	Rhodophyta
Stenogramma interrupta	Rhodophyta

2. SESSILE INVERTEBRATES

TAXON	PHYLUM
Acanthancora cyanocrypta	Porifera
Encrusting sponge spp.	Porifera
Leucilla nuttingi	Porifera
Spheciospongia confoederata	Porifera
Tethya aurantia	Porifera
Abietinaria spp.	Cnidaria
Aglaophenia spp.	Cnidaria
Alcyonium rudyi	Cnidaria
Anthopleura artemisia	Cnidaria
Anthopleura sola	Cnidaria
Anthopleura spp.	Cnidaria
Astrangia lajollaensis	Cnidaria
Balanophyllia elegans	Cnidaria

Corynactis californica	Cnidaria
Halcampa decemtentaculata	Cnidaria
Leptogorgia chilensis	Cnidaria
Metridium senile	Cnidaria
Muricea californica	Cnidaria
Muricea fruticosa	Cnidaria
Obelia spp.	Cnidaria
Pachycerianthus fimbratus	Cnidaria
Paracyathus stearnsi	Cnidaria
Phyllactis spp.	Cnidaria
Plumularia spp.	Cnidaria
Renilla koellikeri	Cnidaria
Urticina lofotensis	Cnidaria
Urticina piscivora	Cnidaria
Cirriformia luxuriosa	Annelida
Diopatra ornata	Annelida
Dodecaceria fewkesi	Annelida
Eudistylia polymorpha	Annelida
Phragmatopoma californica	Annelida
Pista elongata	Annelida
Sabellidae spp.	Annelida
Salmacina tribranchiata	Annelida
Phoronidae spp.	Phoronida
Chaceia ovoidea	Mollusca
Crassadoma giganteum	Mollusca
Mytilus californianus	Mollusca
Parapholas californica	Mollusca
Serpulorbis squamiger	Mollusca
Tubiculous amphipod spp.	Arthropoda
Balanus spp	Arthropoda
Barentsia spp.	Entoprocta
Bowerbankia gracilis	Bryozoa
Bugula californica	Bryozoa
Bugula neritina	Bryozoa
Cellaria spp.	Bryozoa
Celleporina robertsoniae	Bryozoa
Crisia occidentalis	Bryozoa
Diaperoforma californica	Bryozoa
Encrusting bryozoan spp.	Bryozoa
Heteropora pacifica	Bryozoa
Hipporina mexicana	Bryozoa

Membranipora tuberculata	Bryozoa
Phidolopora labiata	Bryozoa
Thalamoporella californica	Bryozoa
Watersipora subatra	Bryozoa
Cucumaria miniata	Echinodermata
Cucumaria piperata	Echinodermata
Cucumaria salma	Echinodermata
Cucumaria spp.	Echinodermata
Eupentacta quinquesemita	Echinodermata
Lissothuria nutriens	Echinodermata
Pachythyone rubra	Echinodermata
Archidistoma psammion	Chordata
Chelyosoma productum	Chordata
Clavelina spp.	Chordata
Euherdmania claviformis	Chordata
Polyclinum planum	Chordata
Pycnoclavella stanleyi	Chordata
Styela montereyensis	Chordata

3. SEA URCHINS

TAXON	PHYLUM
Lytechinus anamesus	Echinodermata
Mesocentrotus franciscanus	Echinodermata
Strongylocentrotus purpuratus	Echinodermata

4. SEA STARS

TAXON	PHYLUM
Dermasterias imbricata	Echinodermata
Orthasterias koehleri	Echinodermata
Patiria miniata	Echinodermata
Pisaster brevispinus	Echinodermata
Pisaster giganteus	Echinodermata
Pisaster ochraceus	Echinodermata
Pycnopodia helianthoides	Echinodermata

5. REEF FISH

TAXON	PHYLUM

Alloclinus holderi	Chordata
Anisotremus davidsonii	Chordata
Aulorhynchus flavidus	Chordata
Bothidae spp.	Chordata
Brachyistius frenatus	Chordata
Caulolatilus princeps	Chordata
Cephaloscyllium ventriosum	Chordata
Cheilotrema saturnum	Chordata
Chromis punctipinnis	Chordata
Citharichthys stigmaeus	Chordata
Cottidae spp.	Chordata
Cymatogaster aggregata	Chordata
Embiotoca jacksoni	Chordata
Embiotoca lateralis	Chordata
Gibbonsia spp.	Chordata
Girella nigricans	Chordata
Gymnothorax mordax	Chordata
Halichoeres semicinctus	Chordata
Heterodontus francisci	Chordata
Heterostichus rostratus	Chordata
Hexagrammos decagrammus	Chordata
Hexanchus griseus	Chordata
Hyperprosopon argenteum	Chordata
Hypsurus caryi	Chordata
Hypsypops rubicundus	Chordata
Leiocottus hirundo	Chordata
Lythrypnus dalli	Chordata
Medialuna californiensis	Chordata
Myliobatis californica	Chordata
Neoclinus blanchardi	Chordata
Ophiodon elongatus	Chordata
Oxyjulis californica	Chordata
Oxylebius pictus	Chordata
Paralabrax clathratus	Chordata
Paralabrax nebulifer	Chordata
Paralichthys californicus	Chordata
Phanerodon furcatus	Chordata
Platyrhinoidis triseriata	Chordata
Rhacochilus toxotes	Chordata
Rhacochilus vacca	Chordata
Rhinobatos productus	Chordata

Rhinogobiops nicholsii	Chordata
Scorpaena guttata	Chordata
Scorpaenichthys marmoratus	Chordata
Sebastes atrovirens	Chordata
Sebastes auriculatus	Chordata
Sebastes carnatus	Chordata
Sebastes caurinus	Chordata
Sebastes chrysomelas	Chordata
Sebastes flavidus	Chordata
Sebastes miniatus	Chordata
Sebastes mystinus	Chordata
Sebastes paucispinis	Chordata
Sebastes rastrelliger	Chordata
Sebastes serranoides	Chordata
Sebastes serriceps	Chordata
Semicossyphus pulcher	Chordata
Sphyraena argentea	Chordata
Squatina californica	Chordata
Stereolepis gigas	Chordata
Syngnathus spp.	Chordata
Triakis semifasciata	Chordata
Xenistius californiensis	Chordata
